# A new role for the nuclear basket network

**DOI:** 10.15698/mic2017.12.604

**Published:** 2017-11-27

**Authors:** Paola Gallardo, Silvia Salas-Pino, Rafael R. Daga

**Affiliations:** 1 Centro Andaluz de Biología del Desarrollo, Universidad Pablo de Olavide-Consejo Superior de Investigaciones Científicas, Junta de Andalucia, Seville, Spain.

**Keywords:** Alm1, TPR nucleoporins, nuclear pore complex, proteasome, kinetochore, fission yeast

## Abstract

Our view of the nuclear pore complexes (NPCs) as gateways between the nuclear and cytoplasmic compartments has been largely expanded in recent years. NPCs have now demonstrated roles in genome regulation and maintenance from single cells to multicellular organisms. Both NPC proteins as well as components of the NPC basket act as dynamic scaffolds for silencing factors, and chromatin and cell cycle regulators. Components of the NPC basket also couple mRNA production and export, and prevent the exit of unprocessed mRNAs from the nucleus. Our recent work describes a novel function of the fission yeast nuclear basket component - the translocated promoter region (TPR) nucleoporin Alm1 - in proper localization of the proteasome to the nuclear envelope. Here we discuss how regulation of proteasome localization to the nuclear envelope by Alm1 is key to maintain kinetochores homeostasis and proper chromosome segregation.

The nuclear envelope (NE) is a bilayer membrane that separates the DNA-containing nucleoplasm from the cytoplasm. Communication through these two cellular compartments is achieved through the nuclear pore complex, a macromolecular channel that transverses the NE. NPCs act as a highly selective gate for the bi-directional exchange of proteins and the export of mRNA from the nucleoplasm. However, a growing body of evidence indicates that both NPCs and NE have a pivotal role in maintaining nuclear organization and thus, they are also essential to maintain genome integrity.

The NPC is composed of multiple copies of roughly 30 different nucleoporins (nups) organized in a channel with eight-fold symmetry. Eight filaments emerge from both the cytoplasmic and nucleoplasmic face of the NPC. The nucleoplasmic filaments are organized in a basket-like structure that projects from the NPC towards the nuclear interior called "Nuclear Basket". The major constituents of the nuclear basket, the TPR nups, are evolutionarily conserved and consist of large (~200kD) coiled-coil proteins that function as a dynamic scaffold for different protein complexes at the NPC.

One of the best-studied roles for TPR nucleoporins is in transcriptional regulation and mRNP export. *Saccharomyces* *cerevisiae* expresses two similar but functionally distinct TPR orthologues, Mlp1 and Mlp2. In *S. cerevisiae,* active transcription of *GAL* genes occurs at the nuclear periphery. The assembly of transcriptional complexes at the nuclear periphery is mediated by the nuclear basket: Mlp1 binds the SAGA (Spt-Ada-Gcn5-acetyltransferase) chromatin-modifying complex, whereas Nup1 interacts with the TREX-2 (Transcription Elongating and RNA Export) complex. Deregulation of any of these elements results in delocalization of *GAL* genes from the nuclear basket and deregulated transcription. This role of Mlp1 does not seem to be restricted to inducible genes, but also affects constitutive ones. Mlp1 interacts with the poly(A) binding protein Nab2, thus recruiting poly(A) mRNAs to the NPC, where Mlp proteins additionally act as a quality control mechanism to retain in the nucleus unspliced or aberrantly processed mRNPs.

Mlps are also involved in transcriptional memory. Inducible genes retain memory of their recent transcriptional activity during intervening periods of repression by creating gene-loops that transiently inhibit transcription. This memory allows rapid recruitment of RNA polII to promoters and faster reactivation of transcription. Mlp1 and Mlp2 by binding to specific DNA sequences at promoters, specifically recruit a different set of regulated genes to the nuclear periphery and are required for transcriptional memory. Recently, Mlp1/2 have been shown to prevent R-loop formation during transcription. The proximity of transcribed genes to the NPC or "NPC-gene gating", dependent on Mlp1/2, avoids R-loop formation likely by facilitating the export of mRNP out of the nucleus. Thus, the nuclear basket works as a platform to coordinate the function of several multiprotein complexes involved in chromatin regulation, transcription regulation, mRNA biogenesis and proofreading and mRNA export.

TPR nucleoporins also act as spatial regulators of the Spindle Assembly Checkpoint (SAC), a mitotic surveillance mechanism that inhibits the metaphase to anaphase transition when kinetochores are not properly captured by spindle microtubules (MTs). The core SAC components Mad1 and Mad2 localize at the NPC during interphase in a TPR/Mlp-dependent manner, and this seems to be evolutionarily conserved as it has been found in* S. cerevisiae, Aspergillus nidulans*, *Arabidopsis thaliana, Caenorhabditis elegans,* *Drosophila melanogaster*, and in human cells. During mitosis Mad1-Mad2 accumulate at kinetochores, where they are essential for SAC activation. The requirement of TPR/Mlp proteins for the localization of Mad1 and Mad2 at kinetochores appears to be less conserved. *A. nidulans* TPR orthologue Mlp1 associates with kinetochores and is required for proper localization and function of Mad1 during mitosis. Human TPR and Megator (Mtor), the *D. melanogaster* TPR counterpart, are part of a fusiform structure called the nuclear matrix, which surrounds the mitotic spindle, and that is a dynamic structural scaffold for key mitotic regulators. Among these mitotic regulators are the component of the SAC Mph1, Mad1 and Mad2. Human TPR- and Mtor- depleted cells show an accelerated progression through mitosis compared to control cells, decreased levels of Mph1, Mad1 and Mad2 at kinetochores, and weakened response to MT depolymerisation, suggesting that TPR and Mtor are required for proper SAC response.

*S. cerevisiae* Mlps are required to anchor Mad1 at the nuclear basket. Mad1 has been shown to cycle between the NPCs and the kinetochores during spindle perturbation in order to elicit the Kap121p transport inhibitory pathway (KTIP). This prevents Kap121-dependent nuclear import of cargos during SAC activation. The deletion of Mlps results in loss of the KTIP. It is thought that phosphorylation on both Mad1 and the Mlps by mitotic kinases regulates the cycling of Mad1 and the KTIP.

TPR nups are highly regulated. For instance, human TPR is phosphorylated at several residues by ERK2, PKA and CDK1 kinases and these phosphorylations are key for the regulation of the differential localization and function of human TPR. Altogether, these studies show that the nuclear basket also works as a dynamic and highly regulated platform that regulates SAC localization and activity and coordinates SAC signalling with transport through the NPC.

*Schizosaccharomyces pombe* has two members of the TPR family of nups, Nup211 and Alm1. Nup211 is essential for cell viability and is required for mRNA export. In a recent study from our lab, we characterize the function of Alm1. We found that Alm1 is required for proper chromosome segregation: the absence of Alm1 resulted in a delay in the metaphase to anaphase transition due to SAC activation and lagging chromosomes during anaphase. Alm1 is required for proper localization of Mad2 to the NPC during interphase, which indicates that this function of TPR nups is also conserved in *S. pombe*. In* alm1*Δ cells Mad2 and Bub1, a key signalling kinase of the SAC, localize properly at kinetochores in normal mitosis and during MT-perturbation, and double mutants between *alm1*Δ and *mad2*Δ or *bub1*Δ showed a negative genetic interaction. This indicated that Alm1 is not required for SAC functionality, but instead SAC function is essential for *alm1*Δ viability. Consistently, we observed altered kinetochore behaviour during metaphase in *alm1*Δ cells, which suggests that in the absence of Alm1 kinetochore capture by spindle MTs is affected and SAC is consequently activated. These results led us to perform a synthetic genetic array (SGA) analysis in conditions that affected kinetochore-MT capture (in the presence of the MT-depolymerizing drug thiabendazole). This genomic approach revealed a strong genetic interaction between *alm1*Δ mutant and mutants in SAC components, ubiquitin-dependent proteolysis, chromatin regulators and kinetochore components.

Kinetochores assemble on the centromere, a specialized chromatin domain characterized by the presence of Cnp1, a centromere-specific histone variant and by the non-canonical nucleosomal complexes formed by CENP-S-T-W-X (SpMhf1, SpCnp20, SpNew1 and SpMhf2). Cnp1 is considered the landmark for kinetochore assembly and directly interacts with CENP-C/SpCnp3 that, in turn, recruits other kinetochore factors such as CENP-L/SpFta1, CENP-K/SpSim4 or the monopolin complex Pcs1-Mde4. Surprisingly, we found that in the absence of Alm1 several kinetochore components were upregulated such as Mhf2, Cnp20, Mde4, Fta1 and especially Cnp3, that showed a two-fold enrichment at kinetochores compared to wild type cells. Cnp3 enrichment was not the result of altered transcription, which led us to the hypothesis that in *alm1*Δ cells Cnp3 degradation was affected.

One of the main cellular mechanisms responsible for protein degradation is an evolutionarily conserved large protease complex termed the proteasome. The proteasome functions in the cytoplasm and also is enriched in the nucleus. In *S. pombe,* the NE protein Cut8 is required to anchor the proteasome to the NE and is also required for proteasome function. Importantly, Cnp1 degradation is regulated locally by the proteasome, which restricts Cnp1 localization to centromeres. Cut8 is also required for Cnp1 degradation, suggesting that proteasome-NE localization has biological significance in the regulation of kinetochore composition.

We provided evidence that Cnp3 is a substrate of proteasomal degradation; Cnp3 accumulated at centromeres after proteasome and Cut8 inactivation. Cnp3 became unstable in the presence of the protein synthesis inhibitor cycloheximide, and ubiquitinated forms of Cnp3 accumulated after proteasome inactivation. Furthermore, Cnp3 decay in the presence of cycloheximide was delayed in *alm1*Δ compared to wild type cells, suggesting that Alm1 is needed for efficient proteasomal degradation of Cnp3. Finally, we found that Alm1 is required for proper localization of Cut8 and the proteasome subunits Mts2 and Mts4 to the nuclear periphery. We hypothesize that Alm1 might directly regulate or stabilize Cut8 localization at the NE, or alternatively, Alm1 might function as scaffold for proteasomal assembly or regulation, which might indirectly impact Cut8-NE localization (Figure 1).

**Figure 1:**
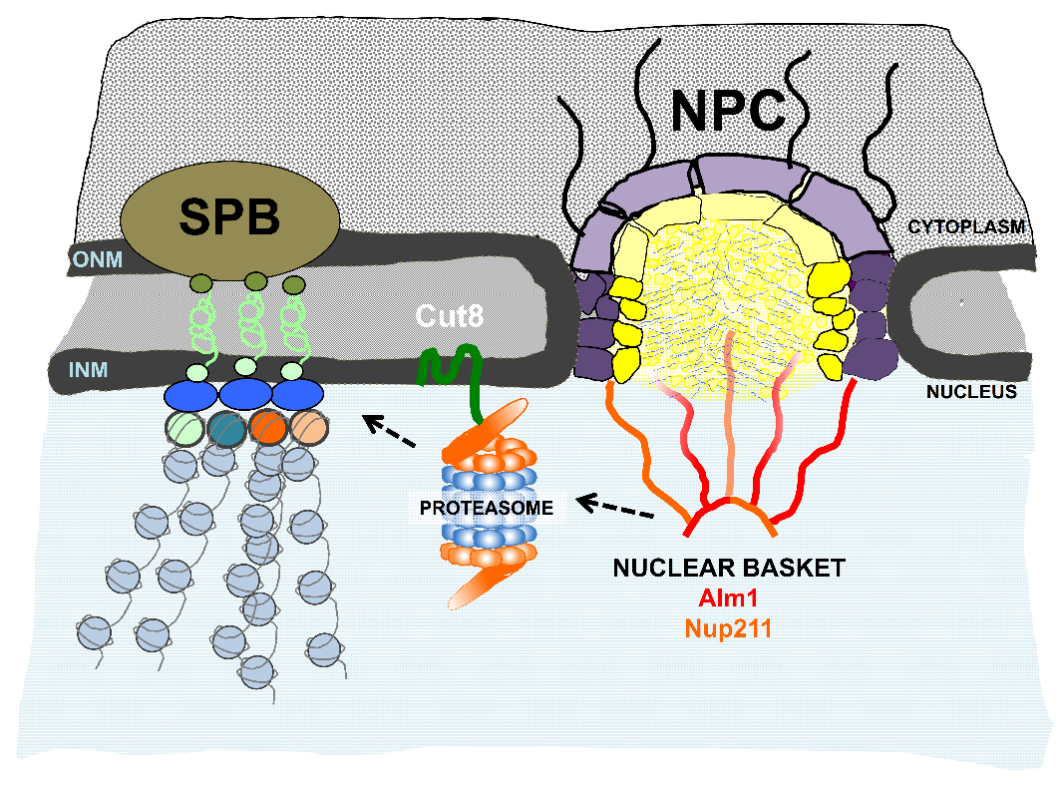
FIGURE 1: Schematic representation of NPC, Centromere/Kinetochore clustering and proteasome localization to the NE. Working model. The fission yeast nuclear basket component Alm1 is required for proper localization of the proteasome and its anchor Cut8 to the NE. Deregulation/delocalization of proteasome from the NE results in unbalanced kinetochore composition, which leads to chromosome missegregation.

The biological role of the proteasome at the nuclear basket remains an open question; however, there is a growing body of evidences that points to a functional relevance of the proteasome at the vicinity of the NE. Mlps interact with Esc1, a NE protein involved in telomere-NE anchoring and silencing, which has also been shown to bind to the proteasome. In *S. pombe*, kinetochores are assembled during the whole cell cycle and during interphase they are clustered and attached to the NE underlying the SPB (Spindle Pole Body, the centrosome equivalent in yeasts). Our study shows that Alm1-dependent proteasome localization at the NE is required for kinetochore homeostasis and places proteasomal regulation as a new function at the network of the nuclear basket.

